# Understanding the Experience of Myotonic Dystrophy. Mixed Method Study

**DOI:** 10.1007/s10897-014-9752-1

**Published:** 2015-02-01

**Authors:** Amy Østertun Geirdal, Inger Lund-Petersen, Arvid Heiberg

**Affiliations:** 1 Faculty of Social Sciences, Oslo and Akershus University College of Applied Sciences, PB 4, St Olavs plass, 0130 Oslo, Norway; 2 Centre for Congenital Neuromuscular Disease, Oslo University Hospital, Rikshospitalet, Oslo, Norway; 3 Department of Medical Genetics, Oslo University Hospital, Rikshospitalet, Oslo, Norway

**Keywords:** Psychological distress, Quality of life, Myotonic dystrophy, Next of kin, Mixed method, Genetic counseling

## Abstract

Myotonic Dystrophy (DM) is a progressive multi-systemic disorder characterized by myotonia and muscle weakness where currently no effective treatment or cure to prevent or delay the disorder exists. This study used mixed methods to examine the experience of living with DM, in patients and their close relatives. Thirteen patients and eight next of kin responded completing Quality of Life and Psychological distress questionnaires in this cross-sectional study, and participating in a semi-structured interview. The findings indicate a higher level of anxiety and hopelessness in next of kin compared to patients, while patients were more depressed. Next of kin reported higher physical, but lower emotional quality of life than patients. Qualitative interviews confirmed the questionnaire findings. The findings from this study may be helpful in genetic counseling. Genetic counselors and geneticists should not only be aware of the burden of being a next of kin, but include discussions about opportunities to minimize the burden in families affected with DM. The findings may be of relevance in counseling for other types of neuromuscular disorders.

## Introduction

Myotonic Dystrophy (DM) is an autosomal dominant, progressive neuromuscular disorder caused by mutations in chromosome 19 (DM1) or more rarely chromosome 3 (DM2) (Harper 2009; Schara and Schoser 2006). DM is the most common adult form of hereditary dystrophic disorders, and most of these patients have a DM1 mutation (Harper 2009; Mankodi 2008). The main features of DM in general are muscular weakness, atrophy and myotonia (Mankodi 2008; Schara and Schoser 2006). Additional multisystem disorder symptoms and signs include cataracts, endocrine, cardiovascular and neurological abnormalities as well as affective changes. Congenital onset and cognitive dysfunction have only been reported in DM1 (Mankodi 2008). DM can present at all ages, however the earlier the DM symptoms appear the more multisystem involvement (Fokstuen et al. 2001).

The DM population has increased owing to improved molecular genetic diagnosis as well as developments in treatment. This has resulted in an increasing number of individuals recognized with long-term health problems. In addition to the health problems, social and psychological demands on the family system can place burdens especially on the immediate family members of persons afflicted by DM, as shown in a study with three groups of muscular dystrophy (Boström and Ahlström 2005a). Our own clinical experience shows that everyday life in a family where one or more members affected by DM1 is often characterized by changes in various routines made necessary by the illness. The family’s ability to deal with the challenges represented by this chronic condition and its effects depend in great part on how family members are able to perceive and integrate DM1 and its influence on their everyday lives through processes of “normalization.”

Adulthood is normally characterized by high levels of activity and productivity, involving processes of gaining educational credentials, starting careers, getting married, starting families, and preparing for and then retirement. However, these normal life processes, including goals and dreams, may become complicated when a chronic disability like DM1 affects one or more family members. The resulting challenges to physical, mental and cognitive functioning may vary, as well as feelings of fatigue, powerlessness and psychosocial consequences (Mankodi 2008). Since DM is a dominantly inherited disorder, on average, one of the parents of the DM affected individual as well as half of her/his siblings and own children are affected by the illness, and impaired functions may affect and be shared with other family members (Hilton-Jones 1997). Increased severity compared to previous generations characterizes DM, in contrast to most other autosomal dominant disorders. Anticipation is almost exclusively maternal. Increasing disability among individuals with DM has been shown to be associated with increased dependency (Nätterlund et al. 2000), and it is documented that the persons affected by DM view the future in terms of the need for more help (Boström and Ahlström 2004). However, many DM affected individuals concentrate on “day to day” functioning and avoid thinking about the future and its demands (Boström and Ahlström 2004).

During the past decade, studies have been published indicating that Quality of life (QoL) in patients with different dystrophic muscular disorders is associated with age of onset, civil status and having children (Boström and Ahlström 2005b), severity and duration of the illness as well as specific emotional functioning (Boström and Ahlström 2005b; Peric et al. 2010). Other aspects of physical functioning, such as gastrointestinal symptoms, have been shown to impact negatively on one’s QoL (Rönnblom et al. 2002) together with sleep problems (Culebras 2005; Dhand and Dhand 2006). Increased need for help is associated with DM patients’ feelings of reduced levels of well-being and higher levels of anxiety and depression (Timman et al. 2010). Compared to healthy relatives and controls, adults with DM are found to have lower scores on QoL (Antonini et al. 2006; Boström and Ahlström 2005b; Kalkman et al. 2005). According to Antonini et al. (2006) personality changes, affective symptoms, and cognitive and emotional deficits, also appear to impact negatively on QoL in DM1 patients.

Depressive or anxiety symptoms, however, have not been found more frequently in the DM group compared either to patients with facioscapulohumeral dystrophy or to healthy controls. On the other hand DM affected individuals score higher in relation to emotional deficits (i.e. monotonous mood states, apathy, and inability to anticipate pleasure) (Bungener et al. 1998).

Among partners/spouses of DM patients lower levels of well-being and higher levels of anxiety were found to be associated with lack of initiative from the patients as well as reduced marital satisfaction (Timman et al. 2010). Next of kin of the affected individual, including partners, parents, children, siblings and more distant relatives experience poorer QoL in comparison to persons living without such burdens (Boström and Ahlström 2005b). On the other hand, in a qualitative study of the next of kin who had two or more relatives (called “double kin”) affected with neurofibromatosis type 1, these individuals reported that, despite their experienced health burden, they saw themselves as healthy and with overall good QoL (Hoxmark 2010).

The main thrust of the research literature thus far supports the notion that being affected with DM gives rise to numerous illness-related problems in everyday life and that individuals affected by this disorder typically face a range of serious challenges to psychosocial functioning including coping, mental health and lower QoL. Less focus has been given the next of kin to the DM affected person.

Most of the studies described above identified their patient group as DM in general and did not distinguish between DM1 and DM2. Myotonic dystrophy type 1 (DM1) and type 2 (DM2) share many of the same clinical features which may be the reason for the lack of distinction. However, DM1 and DM2 have key differences. DM1 is the only form with known cognitive dysfunction and congenital onset, and the multi-systemic effects are typically more burdensome than the muscle weakness. This aspect may lead to occupational and psychosocial limitations affecting coping, management, psychosocial health and quality of life, as emphasized by Mankodi (2008).

Our study focused on the DM1 group. The purpose of this study was to use mixed method (i.e., both qualitative and quantitative methods) to describe the burden of being a next of kin compared to the affected individuals, including their psychological distress and quality of life. The research questions were: 1) How do patients and next of kin to patients with DM1 experience their burden? 2) Do next of kin and the person with DM1 have a similar experience of quality of life and psychological distress? 3) Is there a correlation between experienced QoL and psychological distress? and 4) Is the experienced burden higher in next of kin compared to their affected family member? For genetic counselors and geneticists who meet families affected with DM1, the findings should be of interest and useful.

## Methods

### Design

Our study used a mixed method design. The quantitative component allowed for an examination of possible correlations between QoL and psychological distress and for comparisons between next of kin and patients with DM1. The qualitative component was characterized by in-depth interviews about these issues.

### Participants

Thirty-two individuals with a clinical diagnosis of DM1 confirmed by genetic testing were invited to participate in the study. All were out-patients in different stages of the disorder at the Centre of Congenital Neuromuscular Disorders at Oslo University Hospital/Rikshospitalet. They were invited to include a relative or next of kin who they regarded as important or influential in their lives. Both patients and their identified next of kin were recruited via letters to the patients asking them to inform their relatives or next of kin, about the research. All participants, both patients and their next of kin, signed documents of informed consent. Ethical approval for the study was obtained from the Regional Committees for Medical and Health Research Ethics (REC).

When performing a qualitative study, it is customary to use strategic or purposeful sampling methods to obtain participants who are able to provide rich information (Kvale and Brinkmann 2009; Patton 2002). Due to the relatively small number of the total DM population at the Centre all were invited to participate both in the quantitative and qualitative parts of the study, and thereby ensure as comprehensive and rich information as possible.

### Measures

Demographic questionnaire. Demographic and DM1-related information were collected in both groups, including background information about age, level of education, civil status and working-life status, as well as DM1-related issues such as duration of symptoms, years since the diagnosis, and level of disabilities.

Quantitative questionnaires. To examine Quality of life (QoL) and psychological distress, self-report questionnaires were used. These include overall quality of life (OQoL), well-being (WB), and health-related quality of life (HRQoL) questionnaires, as well as questionnaires measuring psychological distress. Cantril’s (1965) Self-Anchoring Ladder (CL) and Kaasa’s (Kaasa et al. 1988) test (KT) measured OQoL and WB, respectively, while the Short Form 36 (SF-36) (Ware et al. 1996a) assessed HRQoL.

The Cantril’s Ladder (CL) is an overall quality of life questionnaire consisting of one question, “How is your life?” asking respondents to rate their present satisfaction with life on a scale anchored by their own identified values. The response alternatives are 0–10 (0 = worst possible quality of life, 5 = half-way between worst and best, 10 = best possible quality of life) (Cantril 1965). The CL has been shown to have with high validity and reliability (Kolstad 1996).

The Kaasas Test (KT) assesses an individual’s psychosocial experience of well-being (WB), and consists of ten items, five positive and five negative statements (Rating scale: 1 = highest WB to 5 = lowest WB) (Kaasa et al. 1988).

The Health Related Quality of Life (HRQoL) measure Short Form 36 (SF-36) (Ware et al. 1996a) is a questionnaire assessing eight dimensions: physical functioning (PF), role limitation due to physical health problems (RP), bodily pain (BP), general health (GH), vitality (energy/fatigue) (VT), social functioning (SF), role limitations due to emotional problems (RE), and mental health (defined as psychological distress and psychological well-being) (MH). The Physical component scale (PCS) is based on the Physical Functioning, Role limitation due to physical health problems, Bodily Pain, and General Health, while the Mental component scale (MCS) involves Vitality, Social Functioning, Role limitations due to emotional problems, and Mental Health dimensions. The SF-36 has been extensively validated (Ware et al. 1996b).

According to a standard SF-36 scoring, all information is transformed into a scale ranging from 0 (worst) to 100 (best). Based on T-transformations, both PCS and MCS have a mean of 50 and a standard deviation of 10 in the US general population. This is also the case in other populations, including the Norwegian population (Gandek et al. 1998). In these, cases (i.e. severe quality of life below cut-off point that should be considered as determinant of service and treatment) is defined by a score <40 on both the PCS and the MCS (Gandek and Ware 1998; Ware 1996; Ware et al. 1996b).

Psychological distress was examined using The Hospital Anxiety and Depression Scale (HADS) (Zigmond and Snaith 1983) and Beck hopelessness scale (BHS) (Beck et al. 1974). The Hospital Anxiety and Depression Scale (HADS) measures self-reported levels of distress (Bjelland et al. 2002) and consists of 14 items with two subscales, anxiety (HADS-A) and depression (HADS-D): each of these subscales has seven items rated on 4-point Likert-style scale (0 = not present, to 3 = maximally present). The summed score on both the HADS-A and the HADS-D ranges from 0 to 21. Cases of HADS-defined possible anxiety and depression disorder was set at a cut-off score ≥8 based on research findings (Bjelland et al. 2002; Olsson et al. 2005). The psychometric properties of the Norwegian version of the HADS have been found to be excellent (Mykletun et al. 2001).

The Beck Hopelessness Scale (BHS) (Beck et al. 1974) is a 20 item questionnaire designed to measure the degree of hopelessness with item score options of 0 or 1. The BHS summed score ranges from 0 to 20, and the lower the score, the more optimism reported by the respondent. Scores from 9 to 13 indicate moderate hopelessness, while scores of 14 or more imply severe hopelessness.

Qualitative interviews. A semi-structured interview, with an interview-guide, was used to ask patients and next of kin about experiences living with DM1 and how it impacted their mental health and quality of life. The questions explored functioning and physical activities, relation to work, family, network and social life, plans and experienced changes. Further, participants were asked about the experience of the burden of the disorder, and for comparison they were asked what they also thought about the other’s (next of kin and patient, respectively) experience. The questions were based on experience from clinical experience as well as former research. All interviews were carried out at the Centre of Congenital Neuromuscular Disease at Oslo University Hospital/Rikshospitalet, and lasted an average of 60 min for both groups. All identifying information was removed when the interviews were transcribed.

### Data Analyses

SPSS version 18 was used for data handling and statistical analyses. Tests for normal distribution were explored using the Shapiro-Wilk Test. The Shapiro-Wilk Test is appropriate for small sample sizes (<50 samples). A significance value of the Shapiro-Wilk Test > 0.05 indicates that the data are normally distributed. Descriptive statistics were calculated for responses to the demographic questionnaire. All items except the PCS component score were “normally distributed,” and parametric tests were used to compare mean-scores between groups with significance level set at *p* < 0.05. In addition, non-parametric statistic (Mann Whitney U-tests) were employed, due to small samples, without different results. Associations between QoL and psychological distress were measured by bivariate correlations, two sided with significance level set at *p* < 0.01 and using Spearman’s rho due to the small sample size.

## Results

### Response Rates

Fourteen of the 32 patients with DM1 invited to participate in the study agreed to participate, giving a response rate of 44 %. These patients were from nine unrelated families, each consisting of from 1 to 4 members. One patient, however, later withdrew from the study so a total of 13 outpatients (41 %) (6 males and 7 females) took part in the study. In comparison, the group of nonparticipants consisted of 12 males and seven females. Two individuals did not identify next of kin, four relatives brought one common next of kin, and each of the remaining patients included one next of kin, who mostly were spouses, but also one child, one child-in-law, or one parent. All together eight healthy relatives or next of kin without risk for DM1 and 13 affected individuals were included in the study. For some of the patients, it was possible to obtain three generational family histories including affected family members. However, for most of the patients, it was possible to identify affected family members in only two generational family histories.

### Demographics and Disorder-Related Variables

The patients were aged between 18 and 61 years of age (mean = 40.9; SD = 14.4). The mean duration since diagnosis was 10.7 years (SD = 9.3) with a range from 1 to 30 years. Four of the respondents (31 %) had university level educations. In comparison, the group of nonparticipants was aged between 18 and 65 years (mean = 42.4; SD = 11.7), the duration since diagnosis was 9.1 years (SD = 6.2) (range: 3–26 years) with no significant differences between the responders and non-responders. Five participants (40 %) were either employed or in school; the others were on disability leaves or had disability benefits. Four (31 %) reported movement inhibition (i.e., reduced mobility), and 4 (31 %) indicated heart problems as their biggest problems, while the remainder described a combination of these two factors as their biggest problem. One respondent was wheelchair bound (i.e., used an assistive device in daily activities), while seven patients (54 %) perceived themselves as slightly disabled (Table 1). As long as the non-participants did not respond to the invitation we could not measure and compare demographic and disorder related variables other than age and duration since diagnosis.

**Table 1:** Demographics in both groups and disorder related variables in patients

	DM patients (*n* = 13)	Next of kin (*n* = 8)
Age		
Mean (SD)	40.9 (14.4)	41.8 (9.4)
Range	18–61	24–55
Females (*n*/%)	7 (54)	5 (71)
Educational level (*n*/%)		
≤ High school	9 (69)	4 (50)
≥ College/university	4 (31)	4 (50)
Work participation (*n*/%)		
Employee	5 (38)	7 (88)
Disability benefits	7 (60)	1 (12)
Years since diagnosis		
Mean (SD)	10.7 (SD 9.3)	
Range	1–30	
Effect of disorder in daily life (*n*/%)		
Some	3 (24)	5 (63)
Much	5 (38)	2 (25)
Very much	5 (38)	1 (12)
Compare own situation with other (*n*/%)		
Never		4 (50)
Seldom	6 (45)	
Often	3 (24)	4 (50)
Very often	4 (31)	
Biggest problem (*n*/%)		
Movement inhibition	4 (31)	
Heart problems	4 (31)	
Both	5 (38)	
Depending of wheelchair (*n*/%)		
Yes	1 (7)	
No	12 (93)	
Perception of own illness		
Bad	11 (85)	
Good	2 (15)	
Own perception of disability (*n*/%)		
Small?	7 (54)	
No	6 (45)	

### QoL Measures

Compared to their next of kin, the DM1 group reported significantly lower levels on the SF-36 dimensions of physical functioning (PF), general health (GH) and vitality (VT), as well as the component scores for PCS and poorer well-being. Nearly 62 % of the patients reported levels of PCS < 40, corresponding to very poor Health Related Quality of Life. The prevalence of poor PCS was 12.5 % in the next of kin. These results are detailed in Table 2.

**Table 2:** Mean and cut-off scores in Quality of Life and well-being in patients and next of kin

	DM patients	DM next of kin	*P*
	*n* = 13	*n* = 8	
Quality of life measures			
Health related QoL			
SF36			
Physical functioning	52.30 (24. 80)	81.42 (34.84)	0.04
Role physical	59.62 (43.94)	53.57 (44.32)	0.77
Bodily pain	57.15 (22.24)	70.00 (32.98)	0.31
General health	35.54 (16.60)	71.17 (24.11)	0.001
Vitality	30.77 (16.31)	54.29 (29.22)	0.03
Social functioning	84.61 (19.87)	82.81 (29.08)	0.86
Role emotional	30.77 (41.86)	29.17 (45.21)	0.94
Mental health	74.77 (13.00)	73.71 (18.98)	0.89
PCS	37.29 (7.6)	48.02 (10.30)	0.02
PCS cases (n/%)	8 (61.5)	1 (12.5)	0.04
MCS	47.67 (6.74)	44.18 (13.57)	0.44
MCS cases (n/%)	2 (15)	2 (25)	0.50
Overall QoL			
Cantril’s ladder	5.85 (2.30)	7.38 (1.92)	0.12
Experience of well-being			
Kaasa’s test	2.77 (0.72)	2.05 (0.82)	0.05

There were no significant differences in reported levels of overall QoL for patients versus their next of kin (see Table 2).

### Psychological Distress

There were no statistically significant differences in anxiety, depression or hopelessness for the two groups (see Table 3).

**Table 3:** Mean and cut-off scores in psychological distress in patients and next of kin

	DM patients	DM next of kin	*P*
	*n* = 13	*n* = 8	
Psychological distress measures			
HADS-A	5.15 (4.12)	7.75 (4.02)	0.17
HADS-A cases (*n*/%)	4 (31)	4 (50)	0.40
HADS-D	5.00 (2.80)	3.88 (4.49)	0.49
HADS-D cases (*n*/%)	2 (15)	2 (25)	0.61
BHS	12.38 (3.62)	15.13 (4.05)	0.23
BHS cases (n/%)	10 (77)	7 (85)	0.55

### Associations Between Psychological Distress and QoL

As shown in Table 4, levels of anxiety and depression were significantly and negatively correlated with overall QoL and well-being in the patient group. Thus, as anxiety and depression increased, overall Qol and well-being tended to decrease for patients. Levels of hopelessness were also significantly and negatively related to well-being for patients. In the next of kin group anxiety and hopelessness were negatively related to well-being, level of depression was negatively associated with overall QoL, well-being, and health-related Qol. All significance levels were at <0.01 (Table 4).

**Table 4:** Significant correlations between psychological distress and Quality of Life in patients and next of kin

			Psychological distress measures					
			HADS		BHS	HADS		BHS
			Anxiety	Depression	Hopelessness	Anxiety	Depression	Hopelessness
			Patients (*n* = 13)			Next of kin (*n* = 8)		
Quality of life measures	Cantril’s Ladder		−0.707	−0.619			−0.923	
	SF36	PCS						
	Component scales	MCS					−0.658	
	Kaasa’s test	Well-being	−0.720	−0.723	−0.658	−0.902	−0.904	−0.804

All correlations were done two-sided with significant level set at *p* < 0.01

Physical component scale (PCS), contributing on the basis of PF, RP, BP, and GH. Mental component scale (MCS), contributing VT, SF, RE, and MH dimensions

### Qualitative Analysis Results

Both patients and next of kin reported that DM1 had a high impact on daily life due to physical changes, ability and desire to participate in different activities such as work and social life, difficulties in planning, and changed relations and roles in the family. In the analysis the researchers identified three major themes related to psychological distress and experienced quality of life when being in a family characterized by DM1: Expectations about life, Misunderstandings, and a feeling of being on a “Roller Coaster.”

#### Theme 1: Expectations About Life

The most common themes due to psychological distress and reduced quality of life were related to the gap between expectations about life and the reality. For all participants, life had been different than expected with changed plans and current life situations. These differences included for some of the patients, as well as next of kin: not having children, and for those having children reduced ability to follow up the children in their activities; reduced travel or having to quit traveling altogether; social isolation to different degrees; and reduced working ability or being forced to terminate employment. Every participant commented on the differences between their earlier expectations and the reality of their actual lives. Examples from the patients are: “I would love to have kids, and we had decided to have two, but due to the disorder we will have none. I feel guilty for [letting down] my spouse”; “Thoughts and plans are changed”; “My wishes and expectations crumbled”; “I do not fear the future, but I can feel depressed because of the situation”; and “The reduced social relations are worse for my spouse than me.”

Equivalent examples from the next of kin include: “Because my spouse can’t help and share daily activities, all the daily burdens are on my shoulders”; “We have become isolated and lonely”; “We used to be social active, and expected this to continue, but people quit inviting us. Although we have a sick family member, that does not mean that any of us never can participate in social life”; “It is difficult for others to understand, and it may seem impolite not to come after an invitation, but we are never invited anymore”; “Because everything has to be planned very carefully, we can never do anything spontaneous”; “I needed to quit my job due to all the responsibilities in/for the family”; “Of course I am sorry we can’t have children, but I have to understand”; “Life became very different and harder, compared to what I expected”; and “It is tough to be the person in charge all the time, we should have shared the burdens.”

#### Theme 2: Misunderstandings

Almost all patients had an experience of being seen as uncommitted, not to mention lazy. Patients described common and recurrent situations where they wanted to participate, but could not afford to participate as wanted in social life, family activities or work. This was often perceived as lacking will and laziness. Examples include: “I am often misunderstood as, I am seen as uninterested and lazy”; “I have to fight all the time and I try to do small things, but I am not believed [when I say] that I am not able to perform these tasks anymore. It is hard”; “The diagnosis gave an answer, it was not only [my] lack of will or laziness”; and “Even [though I] now have a diagnosis, I very often hear I am lazy.” Common for the patients were feelings of sadness and hopelessness due to these misperceptions.

The next of kin’s perceptions can be characterized through the following examples: “He is always tired and without initiative. It is difficult. I am sure that even if he could participate he does not bother”; “I do not really know if he is as tired as he always says”; “Earlier he took responsibilities for the children, but these days, everything is up to me”; “He is doing very little, and when he is doing something, it is because I have told him [to do it]”; and “I can’t be angry, but I do not look into the future without [a] kind of anxiety.”

#### Theme 3: “Roller-Coaster”

Patients described their situation as being on a “Roller-coaster,” meaning life goes up and down. Life goes up in the sense that they feel they have the energy, and down when they feel they cannot meet expectations and desires, tasks and challenges, and despair over this. The feeling is described in the following quotes: “It is like a roller-coaster, some days I am as in older days, the next I am not able to do anything, and then I can feel depressed”; “I have always felt I have been different, but some days are better than other”; “It is so unpredictable, it is mostly down, but also slightly up”; and “The ups and downs do not increase my fear for the future, but I can sometimes feel depressed.” Next of kin, also, expressed feelings of gladness and joy when something works well, but they were very realistic and described the situation most of the time as “downhill.” The fact that the patients’ condition is progressive and that there at the same time are bright spots in their existence, contribute to the next of kin’s feeling of being on a roller coaster, as well: “On the good days we make plans, but very often they do not come to pass. So, really, we can never plan a thing, everything depends on how it is today”; “It is tiresome not to know how today or the next day will be”; and “I am not depressed about the situation, really, but thinking of the future, I have more anxiety.”

### Mixed Method Analysis Results

Even there was no statistically differences between next of kin and patients due to psychological distress measures, the combination of quantitative and qualitative data analysis identified that next of kin experience the situation as more hopeless and have more anxiety for the future than patients, while patients feel more depressed due to the current situation than next of kin.

When side-by-side comparisons of qualitative and quantitative results were done, next of kin’s orally reported experience of hopelessness is contiguous with the results from Becks Hopelessness Scale (BHS) where higher score level indicate more hopelessness; moreover, individuals with scores greater than 14 may be experiencing severe hopelessness. The BHS conceptualizes positive (optimism) or negative (hopelessness) expectations about one’s short- and long-term future (Beck et al. 1974). It is the latter that was prominent in the next of kin’s comments. In the patients, on the other hand, it seems that their expectations, both according to the short- and long-term future are slightly more positive.

The next of kin also reported a tendency to more anxiety, in interviews as well as in the self-reported questionnaires compared to the patients. Anxiety, as measured by the Hospital Anxiety and Depression scale (HADS) can be seen as a response to a threat (Zigmond and Snaith 1983); in this case a threat of worsening health due to DM1. A vigilant attention to threatening cues is an essential feature of anxiety, this vigilance was seen among the next of kin to a higher degree than among the patients themselves.

Depression is a concept with several meanings, but as measured by the HADS it assesses clinical symptoms due to mild mood disorder, mainly including loss of pleasure response (anhedonia) (Zigmond and Snaith 1983). The patients’ tendency to higher score on HADS than next of kin is consistent with their interview responses according to the three main themes: Expectations about life, Misunderstanding, and The feeling of life as a Roller Coaster.

The relationship between psychological distress and quality of life was emphasized as important of both next of kin and the patients, which is consistent with the findings when measuring the association between psychological distress and quality of life with the self-report questionnaires.

## Discussion

The findings indicated that next of kin have better physical Health Related Quality of Life (HRQoL) and well-being than patients with DM1. In the mixed method analysis the findings between the quantitative and qualitative parts of the study were consistent, as well as affirmativing possible differences. The next of kin had a tendency to report more anxiety and hopelessness, poorer mental HRQoL and less depression than patients, although these differences were not statistically significant. In the qualitative part of the study, however, differences and the tendency of there being differences were emphasized. Several associations between Quality of Life measures and psychological distress were found in both next of kin and patients.

What is new in this study is the finding that the burden due to psychological distress of DM1 seems to be more prominent in next of kin than among the patients themselves. This might point to a heavy burden of care for relatives, both in physical, but particularly in mental health respects. In this study a qualitative interview, as well as self-rated questionnaires were used to explore and measure psychological distress, quality of life and well-being. In addition to the interview, anxiety and depression were measured with the self-rated Hospital Anxiety and Depression Scale (HADS) where the anxiety subscale focused on worry, tension and restlessness (Bjelland et al. 2002). A HADS-defined anxiety disorder is found to be highly correlated with self-rated diagnosis of generalized anxiety disorder. This is characterized by excessive and uncontrollable worry (APA 1994), which is closely related to intolerance of uncertainty (Dugas et al. 2004).

The expressed level of anxiety measured with HADS found in the next of kin may be understood as reflecting the high levels of uncertainty they face in dealing with questions about what will happen with their affected family member as well as the future situations of their families. Because anxiety is a response to threat, increased anxiety in the next of kin can indicate perception of the threat, and can be understood as a natural psychological response. Yet, anxiety can also be seen as a healthy response to avoid a coming danger. The finding that the patients tendency to emphasize anxiety less than their next of kin might possibly indicate that DM1-affected individuals worry less about the progressive nature of the disorder and the uncertainty of their future than do their healthy next of kin. This may also be explained by the findings of Timman et al. (2010) which concluded that the higher level of anxiety in partners and spouses was, among other factors like marital satisfaction, associated with lack of initiative from the patient. The findings might be connected with a possible sample bias given that the 13 patients who responded may have higher functioning than those who declined participation and thereby scored “better” than expected on the surveys and interviews, as well.

Physical illness can cause depression over what is lost according to the discrepancy between expectations regarding life and the reality of life. The patients expressed this fact, which in addition to their feelings of being misunderstood and being on a roller-coaster, may explain why they feel depressed. Even next of kin also had similar feelings, although according to the main themes of the interviews, their feelings often had other expressions. They are not trapped in their own bodies and diagnosis, and depressed by that, but often their depression may come from their cognitions, in particular thinking about the reality of their situation. This might explain the expressed differences in mood between next of kin and patients.

Hopelessness was, in addition to being explored in the interview, measured with the self-rated Beck Hopelessness Scale (BHS) (Beck et al. 1974), for which a higher level of hopelessness indicates more negative expectations about short- and long-term futures (Beck et al. 1974). A realistic future perspective for individuals having a DM1 diagnosis include that the situation will worsen physically, also include a decline in mental/cognitive functioning. With this as a background it is understandable that the next of kin reported hopelessness. Their responses likely confirm the major challenge they experience, that is, living as a healthy spouse or close relative in a family affected by DM1; their responses also indicate a more realistic picture of how the future might turn out. In light of these findings, it seems that the next of kin are aware of and possibly understand the situation better than the patient.

The findings that elevated psychological distress are associated with, and affect quality of life (Qol) and well-being, are consistent with the authors’ clinical experience. On the other hand, living together with a partner has been found to be a buffer against reduced mental health related QoL in other groups living with hereditary disease (Geirdal et al. 2006). For the DM1 patient this might be the case. For the healthy next of kin, however, their partners or close relatives are affected with DM1, and it seems that having an affected partner may not be a buffer against poor mental health related QoL, rather it increases the burden.

It is evident that DM1 affects the whole family, not only the patient. However, it seems that next of kin and patient experience the situation differently due to psychological distress and quality of life. To conclude: The finding that it seems to be a heavier burden to be the next of kin than the patient, depends probably on one’s position in the family. However, it is obvious that next of kin think ahead and see the whole family-situation in a different light than the patients, which might cause distress to a somehow higher level. Our study adds evidence to other findings that being a next of kin appears to carry a greater emotional burden, whereas being affected with DM1 has a greater physical burden.

The results show executive function-limitations of individuals affected with DM1 and indicate how this affects both the affected persons and their next of kin. Several of the problems identified in this study reflect common symptoms of the disorder, and could be treated to provide ways for both patient and caregiver to achieve better mental health, quality of life and coping skills. Such treatments could include stimulants for fatigue, and physical therapy to employ for energy conservation and pain management. In addition to understanding the executive issues and individually addressing them with professionals, treatment in groups would be an intervention with great potential for better psychosocial health. Addressing common problems, challenges and solutions in groups led by professionals, as well as self-help groups, have been shown to be of great importance for the participants (Geirdal 2007; Heap 2005). Extended help in the home primarily in order to relieve necessary everyday tasks where close relatives are a spouse or children, freeing up time for all the family members, as well as informational meetings could be another potential intervention with beneficial outcomes. Individual invited to such information meetings would include the DM1 affected individual, and their next of kin, in addition to persons they wanted to invite. The main objective could be to learn more about the disease with the goal of reducing misunderstandings and the feelings of being on a roller-coaster.

### Study Strengths and Limitations

We used established and validated self-report measures in addition to qualitative interviews. This mixed methods approach may be seen as a strength of the study. All individuals with DM1 known at Oslo University Hospital, Rikshospitalet received invitations to participate in the study together with their next of kin. However, only 44 % accepted the invitation, but one withdrew, so a total of 41 % were included in the mixed method analysis. The high number of dropouts possibly may be attributed to different reasons. Some examples are experiencing oneself as “too sick” and not wanting to meet the health care-team when they do not “need to,” that is, not needing to participate in research projects. Non-participants may feel generally ill, thus lacking the energy to participate or travel; and they may generally be uninterested in participating in a study which further identified them as “affected” which might cause further depressive feelings; and they may prefer to use whatever energy they have for their own prioritized tasks. These reasons may explain the low participation rate and the high number of dropouts. The low response rate could also be understood as part of the burden of the disorder’s costs to everyday life. One example of this involves a single patient who withdrew from the study before it was completed, explaining it was too tough to participate due to poor general health conditions and problems with concentration and persistence.

The study is a cross-sectional study, without the possibility of giving an answer as to whether the results would have changed over time. In addition given the small sample size, the statistical analyses may have lacked sufficient power to detect actual differences. A strength is however the mixed method design which showed consistent and confirming findings between the quantitative and qualitative approaches.

### Practice Implications

There is no effective treatment to prevent or delay DM1. In genetic counseling it is important to be aware of the following factors: DM1 is a disorder affecting the whole family, in particular partners and close relatives in addition to the affected person. The situation includes increased anxiety and hopelessness in next of kin and increased depression in patients, as well as reduced quality of life for both next of kin and patients. The findings indicate that it is of importance to recognize this element when the patient and next of kin come to the genetic services or seek help in general. Further, the genetic counselor and geneticist should not only be aware of the burden of being a next of kin but also include discussions about opportunities to minimize the burden in families affected with DM1. The findings from this study might also be helpful when counseling other groups of neuromuscular disorders; the findings could be translated to other genetic disorders that have a similar neurodegenerative course.

### Research Recommendations

Further research are necessary to explore actions and initiatives recommended herein, and how, and which, such initiatives can help reduce psychological distress and improve quality of life in families affected with DM1. Studies could be done that include samples from other clinics in Norway, and even expanding to other Scandinavian and European countries in an effort to increase the study population.
